# The role of ABCG-type ABC transporters in phytohormone transport

**DOI:** 10.1042/BST20150106

**Published:** 2015-10-09

**Authors:** Lorenzo Borghi, Joohyun Kang, Donghwi Ko, Youngsook Lee, Enrico Martinoia

**Affiliations:** * Institute of Plant Biology and Microbiology, University Zurich, Zollikerstrasse 107, 8008 Zurich, Switzerland; † Pohang University of Science and Technology–Division of Integrative Biosciences and Biotechnology, Pohang University of Science and Technology, Pohang 790-784, Korea

**Keywords:** ABCG transporters, auxin, cytokinins, long distance hormonal transport, phytohormones, strigolactone

## Abstract

Plant hormones (phytohormones) integrate endogenous and exogenous signals thus synchronizing plant growth with environmental and developmental changes. Similar to animals, phytohormones have distinct source and target tissues, hence controlled transport and focused targeting are required for their functions. Many evidences accumulated in the last years about the regulation of long-distance and directional transport of phytohormones. ATP-binding cassette (ABC) transporters turned out to play major roles in routing phytohormones not only in the plant body but also towards the outer environment. The ABCG-type proteins ABCG25 and ABCG40 are high affinity abscisic acid (ABA) transporters. ABCG14 is highly co-expressed with cytokinin biosynthesis and is the major root-to-shoot cytokinin transporter. Pleiotropic drug resistance1 (PDR1) from *Petunia hybrida* transports strigolactones (SLs) from the root tip to the plant shoot but also outside to the rhizosphere, where SLs are the main attractants to mycorrhizal fungi. Last but not least, ABCG36 and ABCG37 possibly play a dual role in coumarine and IBA transport.

## Introduction

**Figure 1:**
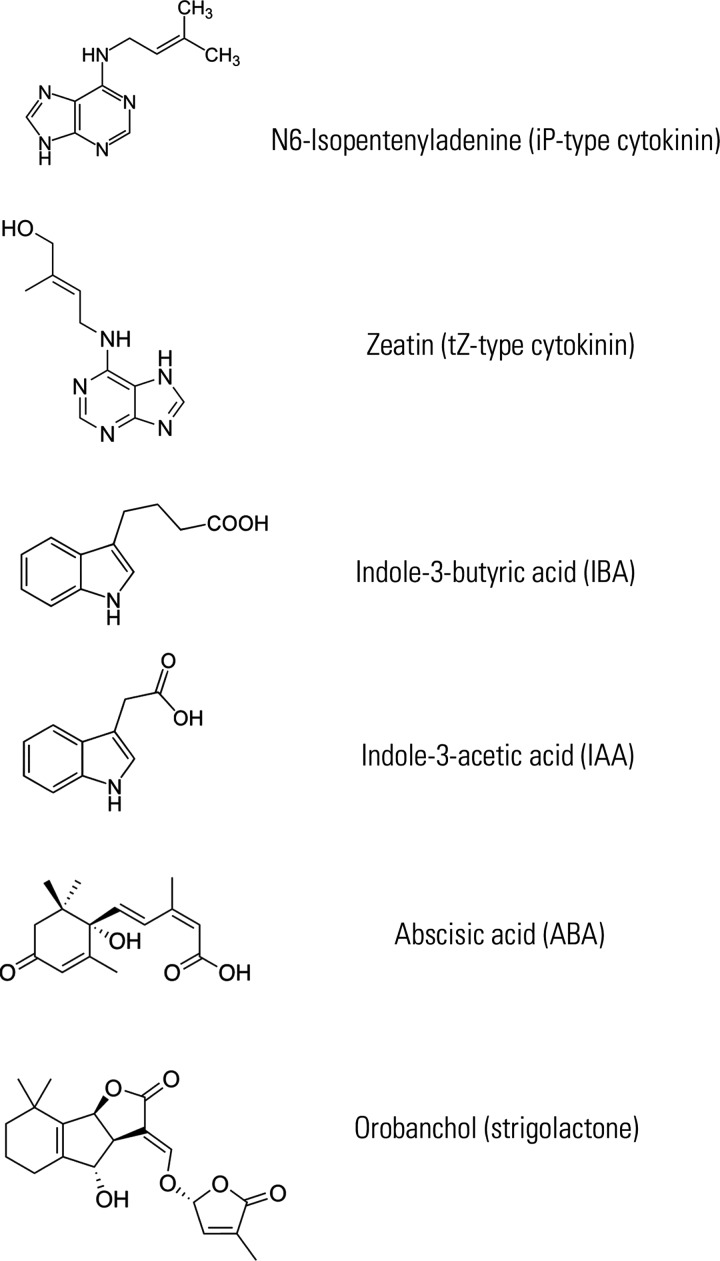
Representation for the chemical structures of all phytohormones discussed

**Figure 2:**
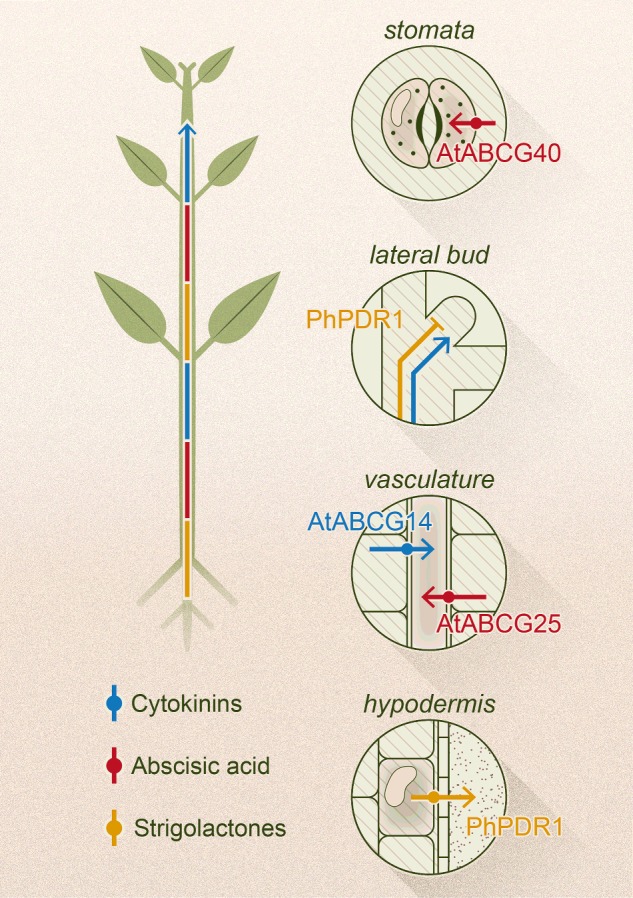
Schematic representation of long-distance transport of phytohormones and their local transport via ABCG proteins in *A. thaliana* and *P. hybrida* (Artwork in collaboration with diogoguerra.com).

Plant hormones (phytohormones) orchestrate plant growth and development in response to endogenous cues and environmental changes. For most phytohormones the importance of transport has been presented or suggested, compatibly with their role of message carriers throughout the plant body. As their animal counterparts, most phytohormones need to be delivered to target tissues far from their biosynthetic sources. The first evidence for a long-distance and directional transport was presented for auxin by Went in 1928 [1]. Later studies revealed that polarly localized pin-formed (PIN) and auxin resistant 1/like aux1 (AUX/LAX) proteins mediate the cell to cell transport of auxin [2] and that ABC (ATP-binding cassette) transporters participate in this process [3]. Physiological and molecular evidence for long-distance transport was also presented ABA, cytokinins and SLs [4–6]. A long-distance signal correlated with jasmonate and mediated by a glutamate receptor-like gene has recently been shown to switch on after wounding [7]. Additionally, it was postulated that jasmonate itself may be transported over long distances to induce wound response [8]. In the case of gibberellins, it is still debated whether they can be transported over long distances. At support of this hypothesis, a recent report showed that gibberellin transport is indeed required for stamen development [9]. During the last years, different families of transporters have been shown to mediate phytohormone transport. These studies revealed that ABC transporters play a central role in exporting and importing auxin, ABA, cytokinins and SLs (see Figure 1 for the chemical structures of the phytohormones here discussed). Since for the main auxin, indoylacetic acid (IAA), so far only ABCB-type transporters have been shown to mediate its transport and several reports have addressed the complex interplay between ABCBs and PINs [10], we will not include this topic in this short review and focus instead on the role of ABCG-type proteins in phytohormone transport (Figure 2).

## Abscisic acid

ABA is known as the hormone playing a main role in biotic and abiotic stress response such as pathogens, salinity, temperature, wounding and water shortage [11]. During drought stress, ABA mediates stomatal closure to prevent water loss [12]. To do this, ABA levels increase dramatically in plants during drought stress [13]. The source of ABA may be diverse. Until about a decade ago it was assumed that ABA synthesized in roots is responsible for the stomatal closure. Work in the laboratory of Professor Grill in Munich showed that when a drought stress was imposed to plant roots, stomatal closure could be observed within a time frame that was not compatible with the speed of the transpiration stream and hence with root to shoot translocation of ABA. Consequently it was shown that after the root has perceived drought stress, an unknown hydraulic signal spreads rapidly to the shoot inducing ABA biosynthesis in the vasculature of the shoot [14,15]. In a series of elegant experiments, Grill and other groups showed that ABA stored in leaf cells or produced *de novo* in the vasculature can be released rapidly to trigger stomatal closure [16]. Free ABA is also released from the ABA storage form ABA-glucoside by two glucosidases, one localized in the endoplasmic reticulum, the other in the vacuole, which hydrolyse the conjugate when plants sense drought stress [17,18]. Furthermore, recent results have shown that guard cells contain enzymes required for ABA synthesis and that upon exposure of leaves to dry air, ABA can be synthesized in guard cells and mediates stomatal closure [19]. Although a consensus existed regarding the fact that ABA has to be exported from cells by a transporter, it was debated whether an importer is required, since under standard conditions the apoplast exhibits a pH of 5.5–6, a condition where a large proportion of ABA is protonated and easily diffuses through membranes. However, it should be kept in mind that water stress leads to a very fast inhibition of the plasma membrane localized H^+^-ATPase and a concomitant increase in the apoplastic pH and hence shifting the ratio of the protonated towards the unprotonated, non-diffusible ABA [20]. Such a change in apoplastic pH allows a plant to control the uptake of ABA in specific cells.

Two reports on ABA transporters were published simultaneously [21,22]. Both are members of the ABCG class. AtABCG25 is a half-size ABC transporter that has been identified in a screen for ABA sensitivity. Germination of *atabcg25* mutant seeds was inhibited by externally applied ABA stronger than the corresponding wild-type. *AtABCG25* localizes to the plasma membrane and is mainly expressed in the vasculature of roots and fruits, but only very weakly in leaves. Additionally it is induced by exogenous ABA treatment in the shoot vasculature. Therefore, *AtABCG25* expression strongly overlaps with ABA biosynthetic enzymes. Indeed, transport experiments using vesicles isolated from insect cells expressing AtABCG25 revealed that AtABCG25 is a high affinity ABA exporter exhibiting a strong substrate preference for the natural (+)-ABA. Further investigations showed that overexpressing *AtABCG25* caused an increase in leaf temperature, indicating a decreased transpiration, which is in line with the role of AtABCG25 as ABA efflux pump and supply of *de novo* synthesized ABA.

The other ABA transporter was discovered using a different approach. Based on the observation that full-size ABCGs can transport terpenoids, the authors postulated that this class of ABC transporters may also be involved in ABA transport. A screen of a large number of ABCG knockout plants revealed that *atabcg40* was impaired in stomatal movement after treatment with exogenous ABA. Using a thermographic camera, the authors could show that leaves of *atabcg40* plants heated up much slower when exposed to ABA, indicating a higher transpiration rate in response to ABA and osmotic stress. Expression of AtABCG40 in yeast and tobacco Bright Yellow-2 (BY-2) cells revealed that AtABCG40 exhibits a high-affinity and stereospecific ABA uptake activity. This was confirmed *in planta* by the observation that ABA responsive genes were up-regulated much slower in *atabcg40* plants than in the corresponding wild-type, when exposed to exogenous ABA.

Based on their initial finding that AtABCG25 acts as an ABA transporter but that the corresponding mutant exhibits only a partial ABA-related phenotype, Kuromori et al. [23] searched for other potential ABA transporter candidates. Using a thermographic camera to screen ABCGs that are closely related to AtABCG25, they identified one mutant that transpired more than the corresponding wild-type. *AtABCG22* is expressed in the aerial part of *Arabidopsis* mainly in guard cells and localizes to the plasma membrane. Interestingly, additive phenotypes were observed for the double knockouts of *atabcg22* with the *open stomata mutant1/snf1-related protein kinase 2 (ost1/srk2)* as well as with mutants for *9-cis epoxycarotenoid dioxygenase3 (nced3)* involved in ABA synthesis. These results suggest that AtABCG22 has an ABA-related phenotype, but does not act directly as an ABA transporter. In line with this hypothesis, the authors could not see any AtABCG22-mediated ABA transport activity when expressed heterologously in various systems.

To investigate the role of the different ABA transporters and additional ABA related genes in respect to a sudden reduction in high humidity, increase in CO_2_ or exposure to ABA, Merilo et al. [24] performed gas exchange experiments. The sole clear ABA-related phenotype was observed with the *atabcg22* mutant when plants were transferred to dry air. Although after 60 min stomata were as closed as in the wild-type, the closure kinetics in the mutant was strongly delayed. No significant difference was observed when plants were exposed to ABA, although a slight change in stomatal kinetics was also observed in *atabcg40*. The lack of clear mutant phenotypes under these conditions can be explained with the fact that (i) AtABCG25 is an ABA exporter and therefore spraying ABA will not significantly change the AtABCG25-dependent apoplastic ABA content in the mutant; (ii) spraying will very probably not affect apoplastic pH and hence mainly result in increasing ABA diffusion; (iii) the function of AtABCG22 remains elusive and cannot be deduced from the experiments performed in this study. Furthermore, it should be mentioned that it is likely that other ABC proteins act as ABA transporters and that members of other transporter families, such as ABA-importing transporter1 (AIT1)/nitrate transporters (NRTs) and multidrug and toxin efflux transporters (MATEs) have also been reported to act as ABA transporters [25–27].

Besides stomatal control, ABA plays an important role also in germination and plant tolerance to pathogens. The ABA transporter mutants reported so far are all partially impaired in germination. For *Arabidopsis*, it has been well-documented that germination is inhibited by a continuous delivery of *de novo* biosynthesized ABA from the endosperm towards the embryo and by uptake into the embryo [28]. However, which ABA transporter fulfils which step can be only hypothesized from its location and transport direction. A detailed analysis is therefore needed to answer this question.

*Pseudomonas syringae DC3000* (Pst DC3000) secretes coronatine, a virulence factor that forces stomata to reopen when plants are attacked by this pathogen. Furthermore, Pst DC3000 leads to an up-regulation of the ABA signalling pathway, which in turn suppresses the salicylic acid-mediated resistance. Using a genome wide association mapping, Ji et al. [29] identified AtABCG16 transporter as a candidate gene implicated in defence against Pst DC3000. This ABC transporter is highly up-regulated by ABA and knockout plants were more susceptible to ABA. Interestingly, *atabcg16* knockouts were also more susceptible to Pst DC3000. The data indicate that AtABCG16 could be an ABA exporter; however, this has to be proven as well as the exact mechanism how the increased sensitivity to pathogens is linked to the increased sensitivity to exogenously applied ABA. Furthermore, it has to be mentioned that ABCG16 has also been reported to be required for the synthesis of an intact pollen cell wall [30]. Whether AtABCG16 is implicated in the transport of such diverse substrates as ABA and components of the pollen wall or whether the different phenotypes observed are mediated by one multifunctional substrate has to be addressed in the future.

## Cytokinins

Cytokinins are phytohormones involved in regulating a myriad of physiological and developmental processes, including shoot apical meristem formation, cambial growth, cell differentiation and nodulation to mention just a few [31].

Until recently it was assumed that cytokinins are synthesized in the root and transported to the shoot via the xylem [32,33]. However, the discovery that cytokinin biosynthesis genes are also present in the aerial part lead to a new view of cytokinin biosynthesis and transport [34]. The present knowledge, based on grafting experiments and detection of cytokinins in xylem and phloem saps, strongly supports the view that the trans-zeatin cytokinins (tZ-type) are transported from the root to the shoot via the xylem, whereas N6-(D2-isopentenyl) adenine cytokinins (iP-type) are transported from the shoot to the root via the phloem [35]. Long-distance transport of cytokinins is a crucial step for cytokinin-mediated regulation of the plant growth and development. For instances, shoot-derived iP-type cytokinins regulate vascular patterning in the root meristem of *Arabidopsis* [36], as well as nodulation in the *Lotus japonicas* roots [37], whereas the root-derived *t*Z-type cytokinins promote shoot growth [38].

Recently, two publications reported highly similar results about the discovery of a cytokinin transporter involved in the transfer of cytokinin from the root to the shoot [5,39]. In both cases the authors performed *in silico* analysis on ABCG transporters of unknown function. In one case, the authors first focused on mutants of ABC transporters exhibiting a strong phenotype possibly related to a specific substrate; in the other the authors investigated whether an ABC transporter was strongly co-expressed with the cytokinin synthesizing genes *adenylate isopentenyltransferase3 (IPT3)* and *cytochrome p450 monooxygenase (CYP735A2)* in the root. Consequently, in the first case the authors deduced from the mutant phenotype that AtABCG14 could be involved in cytokinin transport whereas in the second they identified AtABCG14 as a gene highly co-expressed with cytokinin biosynthesis and hence putatively involved in cytokinin transport. Mutants in *AtABCG14* had much smaller rosette leaves and shorter and thinner flower stems than in the wild-type. Furthermore, the number as well as the size of xylem and phloem cells were strongly reduced in the mutant. Spraying with *t*Z rescued the phenotype in *atabcg14* knockout plants, supporting the idea that AtABCG14 could mediate root to shoot translocation of *t*Z-type cytokinins. Determination of cytokinin contents in roots and shoots revealed that *t*Z-type cytokinins levels were strongly reduced in shoots but increased in roots of the *atabcg14* mutant plants. In contrast, iP-type cytokinin levels were increased both in *atabcg14* roots and shoots. Since former studies showed that cytokinins are transported within the xylem, Ko et al. [39] measured cytokinin levels in the xylem of the wild-type and the mutant plants. The authors observed that within the xylem mainly *t*Z-type cytokinins are transported and that the concentration in the xylem is reduced by ∼90% in *atabcg14* plants. Finally, both laboratories performed loading and transport studies at the whole plant level by applying labelled *t*Z cytokinins to the roots. Both reports showed that *abcg14* mutants transported much less of this cytokinin to the shoot, which is in line with the other results and underlines the role of AtABCG14 as a root localized transporter responsible for the allocation of *t*Z cytokinin to the shoot.

Former studies using grafting experiments of multiple biosynthesis mutants concluded that transport of cytokinins from the root to the shoot is not required for shoot development. In contrast, the studies on AtABCG14 show that the wild-type shoot grafted on the *atabcg14* mutant root exhibited growth retardation. This result shows that the allocation of *t*Z-type cytokinins by AtABCG14 to the shoot is a prerequisite for the correct development of this organ. The discrepancies between the two observations is very probably due to the fact that if a wild-type shoot is grafted on biosynthesis mutant, iP-type cytokinins are transported via the phloem to the root, where they are converted into *t*Z-type cytokinins and reallocated to the shoots via AtABCG14.

Several questions are still open regarding long-distance transport of cytokinins. It is likely that AtABCG14 is the major transporter for the transfer of cytokinins from the root to the shoot, but it cannot be excluded that one or several other transporters participate in this process. A more striking question is how cytokinins are loaded to the phloem to be transferred to the root. This transport process has not only been shown to be important for the development of roots but also for the correct establishment of nodules in nitrogen fixing plants [37].

## Strigolactones

SLs have been originally characterized as germination stimulants of parasitic weeds [40] and for several decades it was questioned why plants excrete compounds that are potentially dangerous for their survival. It was only a few years back that Akiyama et al. [41] discovered that SLs are important plant signalling compounds, leading to hyphal branching of mycorrhizal fungi and hence promoting into soil the plant-fungal mutualistic symbiosis known as mycorrhiza. Three years later, two laboratories [42,43] discovered that the long sought compound co-involved in the inhibition of shoot lateral branching was also a SL. These important results attracted much interest and during the last years a large amount of new insights on SL structure, biosynthesis, signalling and function were published [44]. Grafting studies mostly carried on by Domagalska and Leyser group [45] on *Arabidopsis thaliana* indicated that SLs are synthesized both in shoots and roots by shared players: two carotenoid cleavage dioxygenases (CCDs; *CCD7* and *CCD8*) and a plant-dependent number of cytochrome P450 mono oxygenases (*more axillary growth1 (MAX1)* and *MAX1-like*). However, when wild-type roots were grafted on a SL biosynthesis mutant shoots, the root-stock could supply SL to the scion and complement for the mutant phenotype. From these studies it was deduced that SLs have to follow two transport routes: on one side they have to be exuded from the roots to induce hyphal branching, on the other they are transported shoot wards to regulate the shoot architecture. Based on the observations that ABCG transporters were shown to transport terpenoids [46] and that this class of proteins has been postulated to be involved in biotic and abiotic stress responses [47], Kretzschmar et al. [48] hypothesized that the SL transporter could be a member of this ABC family. Using *Petunia hybrida* as a model system they screened for root-expressed ABCGs that are up-regulated by phosphate starvation, a condition known to induce SL excretion [49] and by GR24, a synthetic SL, since many ABCs are known to be up-regulated by their substrates. One of the candidate genes, petunia *PDR1*, fulfilled these requirements and was further analysed.

Kretzschmar et al. [48] showed that PDR1 was localized in the plasma membrane. At the tissue level PDR1 was expressed in root tips, in the hypodermal passage cells (HPCs), as well in proximity of the root vasculature and in nodes of the stem, close to the axillary buds. Special attention was paid to HPCs, since these were known to be the entry point for mycorrhizal fungi [50]. As petunias carry the natural occurring *defective Transposable element petunia hybrida 1 (dTph1)* [51], the authors searched for a knockout mutant carrying a transposon insertion in *PDR1* and showed that this mutant released less orobanchol, the most abundant SL synthetized in petunia, whereas the content within the root was not altered. Furthermore, root exudates collected from *pdr1* mutant plants were much less efficient in inducing hyphal branching and germination of the parasitic weed orobanchae. In order to test whether PDR1 could be indeed a SL exporter, the authors expressed petunia PDR1 in the far-related species *A. thaliana*, a plant exhibiting a low endogenous SL-exudation activity [52] and where no SL functional homologue is isolated, yet. *Arabidopsis* plants overexpressing PDR1 were more tolerant to supplements of the synthetic SL GR24 and when pre-loaded with radioactively-labelled GR24 excreted by far more of this SL than the corresponding wild -type. These results confirmed that petunia PDR1 was a SL transporter. Above ground, *pdr1* petunia mutants exhibited a stronger shoot lateral branching than the corresponding wild-type, although the phenotype was clearly weaker than what observed for biosynthesis mutants. Similar observations as shown in Kretzschmar et al. [48] were recently reported by Xie et al. [53] for tobacco plants. Here too, expression of NtPDR6, the tobacco closest homologue to petunia PDR1, was observed in HPCs. Furthermore, silencing of *NtPDR6* resulted in plants with a bushy phenotype.

A recent study aimed to investigate in more detail how SLs are transported from their site of synthesis in the root tip shootwards and into the soil. To answer this question Sasse et al. [54] produced plants expressing a GFP–PDR1 fusion construct and determined its localization. PDR1 exhibited a cell-type specific and asymmetric localization in different root tissues. In root tips, *PDR1* was co-expressed with the SL biosynthetic gene *decreased apical dominance1 (DAD1/CCD8)* and it was localized at the apical membrane of root hypodermal cells, presumably mediating the shootward transport of SL. Above the root tip in HPCs, PDR1 was present in the outer-lateral plasma membrane, compatible with its postulated function as SL exporter from root to soil. Transport studies using radiolabelled GR24 supported the localization studies, since the *pdr1* mutant displayed impaired transport of SLs out of the root tip to the shoot as well as into the rhizosphere. Interestingly, the biosynthesis gene *DAD1* was strongly down-regulated in the root tips of *pdr1* mutant plants, indicating a feedback regulation and cross-talk between SL biosynthesis and transport. Furthermore, in plants overexpressing PDR1, the protein levels of the auxin carrier PIN1 were strongly down-regulated in the stele of the root tip whereas the auxin carrier PIN2 was not affected or in the contrary even induced. These results showed how tightly SL and auxin transport are interlinked, as already suggested by previous works, where SLs were exogenously applied to root and shoots of *Arabidopsis* [55–57].

## Auxigenic compounds

In order to identify components of auxin homoeostasis Fujita and Syono [58] performed a screen with the auxin transport inhibitor N-1-naphtylphtalamic acid (NPA) and identified a mutant that is hypersensitive to this drug and affected in above- as well in below-ground development. In order to better understand the mechanism behind this hypersensitivity, Ruzicka et al. [59] mapped the *polar auxin transport inhibitor sensitive1 (pis-1)* mutation and found that the associated gene was *AtABCG37*. The authors showed that the *pis-1* mutant is also sensitive towards natural auxinic compounds such as indole 3-butyric acid (IBA) and that this ABC transporter is localized in the outer-lateral plasma membrane of root epidermal cells. Since a similar localization had been reported for the close homologue *AtABCG36* [60], the authors produced double knockout mutants and showed the auxinic hypersensitivity was even stronger in these plants. Finally they provided direct proof for the transport activity by showing that protoplasts isolated form T-DNA insertion mutants excreted less IBA, whereas *Saccharomyces cerevisae* or HeLa cells expressing this ABC transporter retained more.

Two later studies with *AtABCG37* provided convincing evidence that this ABC transporter is also important for iron nutrition [61,62]. Based on co-expression studies and previous knowledge on iron nutrition regulation in plants, the authors hypothesized that *AtABCG37* may be involved in the excretion of phenolic compounds. Indeed, Fourcroy et al. [61] could show that *AtABCG37* was required for the secretion of coumarine compounds that can complex iron and hence supply iron to plants under conditions of scarce iron availability.

It should be considered that a great variety of microorganisms is associated with the plant root surface and that auxinic compounds may act as signal- or precursors of signal molecules for these organisms too [63]. Therefore, the question arises whether AtABCG37 plays a dual role in coumarine and IBA transport.

A targeted analysis of IBA in root exudates will reveal whether this compound is found in root exudates.

## Conclusion and outlook

In this review we concentrated on transport processes mediated by ABCG proteins. However, it should be mentioned that, for ABA as well as for cytokinin, transporters of other protein families have been also shown to mediate their transport. In the case of ABA, NRTs have been identified as ABA importers. NRT1.2 knockout plants exhibit a higher sensitive towards ABA in germination and inflorescence stomata closure [4]. A MATE transporter has also been shown to mediate ABA transport. In this case, ABA is effluxed from cells. Interestingly, despite the fact that so many transporters have been reported to be involved in ABA transport, all single knockouts exhibit an ABA-related phenotype. Several of these phenotypes are rather weak, indicating that the different transporters work in parallel and that they all exhibit a role in ABA transport and are required for an efficient signal transduction. Production of multiple knockouts will help to elucidate how these transporters collaborate and interact. Furthermore, more detailed expression data are required to figure out whether some of these transporters which are expressed in the same tissue are also localized in the same cell type.

In the case of cytokinins, purine permeases (PUPs) and equilibrative nucleoside transporters (ENTs) have been shown to mediate cytokinin uptake into cells. Since AtPUP1 is highly expressed in the epithelium of hydathodes and stigma surface of siliques, AtPUP1 was suggested to be involved in the import of cytokinins from xylem sap into the cells [64]. In contrast, AtPUP2 and AtENT6 are mainly expressed in the vasculature, indicating that the transporters may mediate phloem loading of cytokinins [34,64]. However, the physiological roles of these transporters have so far not been characterized in detail. Since *Arabidopsis* harbours 21 and 8 members of PUPs and ENTs respectively, an extended study of these groups of transporters might reveal other cytokinin transporters.

We predict that there will be many more discoveries of plant hormone transporters belonging to ABC families and other transporter families. Furthermore, we predict that scientists will find interactions between hormone transporters influencing each other's activity and expression, thus forming a complex network of cross-talks.

## Abbreviations

ABA: abscisic acid
ABC: ATP-binding cassette
CCD: carotenoid cleavage dioxygenase
DAD1: decreased apical dominance1
ENT: equilibrative nucleoside transporter
HPC: hypodermal passage cell
IBA: indole 3-butyric acid
MAX1: more axillary growth1
PDR1: pleiotropic drug resistance1
PIN: pin-formed
PUP: purine permease
SL: strigolactone
